# First Insights into the Draft Genome of *Clostridium colicanis* DSM 13634, Isolated from Canine Feces

**DOI:** 10.1128/genomeA.00385-16

**Published:** 2016-05-19

**Authors:** Anja Poehlein, Tobias Schilling, Udhaya Bhaskar Sathya Narayanan, Rolf Daniel

**Affiliations:** a Genomic and Applied Microbiology & Göttingen Genomics Laboratory, Institute of Microbiology and Genetics, Georg-August University, Göttingen, Germany; b Members of the Applied Bioinformatics in Microbiology Course of the Microbiology and Biochemistry MSc/PhD program, Georg-August University, Göttingen, Germany

## Abstract

*Clostridium colicanis* DSM 13634 is a strictly anaerobic, rod-shaped, and spore-forming bacterium. It produces acids from common sugars such as glucose and fructose. The draft genome consists of one chromosome (2.6 Mbp) and contains 2,159 predicted protein-encoding genes.

## GENOME ANNOUNCEMENT

*Clostridium colicanis* is an anaerobic, rod-shaped, and spore-forming bacterium (1). *C. colicanis* was isolated from the feces of a male Labrador dog (1). A comparative 16S rRNA gene analysis showed that the bacterium forms a distinct subgroup within the *Clostridium* rRNA group I. *C. absonum*, *C. baratii*, and *Eubacterium multiforme* were the phylogenetically closest relatives of *C. colicanis*. These organisms are also typically found in fecal samples (1–3).

Chromosomal DNA of *C. colicanis* DSM 13634 was isolated using the MasterPure complete DNA purification kit (Epicentre, Madison, WI, USA). From the extracted DNA, Illumina shotgun paired-end sequencing libraries were generated, and sequencing was performed with a MiSeq instrument and a MiSeq reagent kit version 3, as recommended by the manufacturer (Illumina, San Diego, CA, USA). Quality filtering using Trimmomatic version 0.32 (4) resulted in 2,106,860 paired-end reads. The assembly was performed with the SPAdes genome assembler software version 3.6.2 (5). The assembly resulted in 83 contigs (>500 bp) and an average coverage of 164-fold. The assembly was validated and the read coverage determined with QualiMap version 2.1 (6). The draft genome of *C. colicanis* DSM 13634 comprises a single chromosome (2.6 Mbp) with an overall GC content of 31.18%. Automatic gene prediction and identification of rRNA and tRNA genes was performed using the software tool Prokka (7). The draft genome contained 14 rRNA genes, 119 tRNA genes, 2,159 protein-encoding genes with function prediction, and 494 genes coding for hypothetical proteins. Interestingly, two CRISPR repeats followed by downstream CRISPR/Cas cluster regions were found. However, putative prophage regions were not detected in the draft genome sequence.

*C. colicanis* DSM 13634 can utilize a large number of substrates (1). It produces several different acids from cellobiose, fructose, galactose, glucose, lactose, maltose, mannose, ribose, and sucrose (1). The formed acids could comprise lactate, acetate, and formate. Correspondingly, putative genes coding for lactate dehydrogenase, phosphotransacetylase, acetate kinase, and formate acetyltransferase were present in the genome of *C. colicanis*. Moreover, mannitol and sorbitol are utilized, but only nonacidic metabolic end-products are formed (1). Genes encoding phosphotransferase systems for the substrates glucose, cellobiose, mannitol, mannose, sorbitol, and fructose were found in the genome. Additionally, genes coding for a galactitol phosphotransferase system were present. As described by Greetham et al. (1), nitrate is reduced to nitrite by *C. colicanis*. We found genes encoding nitrite reductases NasAB (nitrate to nitrite) and NirA (nitrite to ammonia).

### Nucleotide sequence accession numbers. 

This whole-genome shotgun project has been deposited at DDBJ/EMBL/GenBank under the accession number LTBB00000000. The version described here is the first version, LTBB01000000.
